# Is There a Connection Between Gut Microbiome Dysbiosis Occurring in COVID-19 Patients and Post-COVID-19 Symptoms?

**DOI:** 10.3389/fmicb.2021.732838

**Published:** 2021-09-17

**Authors:** Kai Hilpert, Ralf Mikut

**Affiliations:** ^1^Institute of Infection and Immunology, St George's, University of London, London, United Kingdom; ^2^Institute for Automation and Applied Informatics (IAI), Karlsruhe Institute of Technology (KIT), Karlsruhe, Germany

**Keywords:** gut microbiome, COVID-19, long COVID-19, post acute COVID-19, SARS-CoV-2 gut infection

## Introduction

According to WHO, currently 215 countries/areas/territories report a total of more than 176 million confirmed COVID-19 cases and 3.8 million deaths (June 18, 2021). SARS-CoV-2, the causative agent of COVID-19, does not impact only the respiratory system but also the various organs in the body. It can directly or indirectly affect the pulmonary system, cardiovascular system (including heart failure), renal system (including kidney failure), hepatic system (including liver failure), gastrointestinal system, nervous system, and/or various systems, leading to shock and multi-organ failure (Zaim et al., 2020). In consequence, comorbidity in these systems leads to a higher risk for a severe disease progression.

## Post-Acute/Chronic COVID-19

In 2003, there was an outbreak of SARS-CoV-1, and follow-up data showed that, after several years, in about 40% of the patients, long-term/chronic symptoms persisted, for example, psychiatric illness (post-traumatic stress syndrome, depression, somatoform pain disorder, and panic disorder), chronic fatigue, and reduced pulmonary function (Lam, 2009; Ngai et al., 2010). Recently, similar trends of long-term symptoms have been reported for patients infected with SARS-CoV-2. The definition is still evolving as is the name, for example, long COVID-19, COVID-19 long hauler, post-COVID-19, post-acute COVID-19, and chronic COVID-19 are used to describe symptoms from patients with acute COVID-19 that last longer than 4 or 12 weeks and are not attributable to alternative diagnoses. The studies investigating this phenomenon vary in their methods, spanning from simple questionnaire or telephone interviews to physical examination, chest scan, and blood markers. Further varying parameters are the inclusion criteria, the timing of questioning/examination after recovery, age, and severity of cases. Many studies also have a rather small number of participants and therefore need to be looked at with some caution. Despite these limitations, all studies that we are aware of point in one direction: a large percentage of patients, according to a meta-analysis including 47,910 patients, about 80%, developed at least one chronic symptom (Lopez-Leon et al., 2021).

A recent study from China looked at the health status of 1,733 COVID-19 patients (age 47–65 years) 6 months after their discharge from the hospital. They were interviewed with a series of questionnaires for evaluation of symptoms and health-related quality of life; they also underwent physical examination and a 6-min walking test and received blood tests (Huang et al., 2021). From these patients, 76% showed at least one symptom that did not resolve in 6 months. The main symptoms were fatigue and/or muscle weakness (63%), sleep disturbance (26%), dyspnea (23%), anxiety/depression (23%), hair loss (22%), loss of test/smell (7–11%), chest pain (5%), and diarrhea (5%). Patients who had more severe symptoms during their hospital stay had consequently more severe impaired pulmonary diffusion capacities and abnormal chest imaging manifestations. Several smaller-scale studies in France (Garrigues, 2020; Carvalho-Schneider, 2021), UK (Arnold et al., 2021; Halpin, 2021), Italy (Carfì et al., 2020), Spain (Moreno-Pérez et al., 2021), Mediterranean (Moreno-Pérez et al., 2021), and USA (Chopra et al., 2021) showed similar results [for an overview, see Nalbandian et al. (2021)]. In these studies, between 32.6 and 87.4% of the patients reported that at least one symptom had not resolved after COVID-19. The main symptoms were fatigue (34.8–64%), joint pain (4.5–27.3%), sleep disturbance (24–30.8%), dyspnea (11.1–43.4%), loss of taste/smell (10.8–21.7%), cough (2.1–21.3%), headache (1.8–17.8%) hair loss (20%), chest pain (10.8–21.7%), and diarrhea (0.9–10.5%). A meta-analysis including 47,910 patients followed up at 14 to 110 days post-COVID-19 showed that they present very similar results: 80% (95% confidence interval, CI: 65–92%) of the patients developed one or more long-term symptoms. The most common symptoms were fatigue (58%), headache (44%), attention disorder (27%), hair loss (25%), and dyspnea (24%) (Lopez-Leon et al., 2021). Moreno-Pérez et al. showed in a study with a systematic assessment 10–14 weeks after disease onset of 277 patients (age 42–67.5 years, 65.7% with severe illness) that at least one chronic COVID-19 symptom was detected in 50.9%. Importantly, for the patients experiencing a chronic COVID-19 symptom, the life quality deteriorated by a median of nine points according to the EuroQol Visual Analog Scale (Moreno-Pérez et al., 2021).

We believe that it is urgent to define a term describing these symptoms with a clear indication of time after onset of diseases and method of diagnosis, for example, chronic COVID-19 syndrome (CCS), in order to perform more comparable systematic studies not only to gain understanding of the underlying causes but also to develop targeted therapies. In our opinion, important are studies that follow patient groups (severe, hospitalized but not severe, and mild) with a range of diagnostic procedures in order to understand the progression and regression of symptoms. For more than 170 million confirmed cases plus the unconfirmed cases, a high percentage will face CCS, and despite most of them being categorized as mild in medical terms, fatigue, joint pain, headache, anxiety, depression, and dyspnea will lead to a reduced quality of life. Providing curative solutions, not symptom management, should be the aim of further research in this area.

## SARS-CoV-2 and the Human Gut

The gastrointestinal system is vulnerable to infections with SARS-CoV-2. SARS-CoV-2 requires angiotensin I-converting enzyme 2 (ACE2) as a receptor to enter a human cell. In addition, host proteases are required to prime the spike protein, especially transmembrane serine protease 2 (TMPRSS2) (Hoffmann et al., 2020). Hikmet et al. showed that the expression of ACE2, as determined by transcriptomics by three different independent consortia, is very high in the small intestine, 120 consensus normalized expression units (NX), and in the colon and duodenum, both about 50 NX, compared to 0.8 NX in the lung (Hikmet et al., 2020). The data was supported by immunohistochemistry on human tissue, showing the high expression of ACE2 protein in the enterocytes and crypt cell of the duodenum and small intestine and in the enterocytes of the colon. The expression in the lung was also minimal (Hikmet et al., 2020). Using RNAseq, the expression of TMPRSS2 was determined to be high in the small intestine, colon, stomach, and esophagus; however, it was also high in the lungs (TMPRSS2 Gene—GeneCards | TMPS2 Protein | TMPS2 Antibody). The co-expression of ACE2 and TMPRSS2 in esophageal upper epithelial cells, glandular cells, and cells from the ileum and colon was confirmed by single-cell transcriptomic analysis (Zhang et al., 2020). Because of the independent methods, the data is very robust, and the digestive tract, based on the receptor and the protease levels, is an “ideal” place for SARS-CoV-2 infection to occur. It is therefore rather surprising that, according to a meta-analysis comprising 4,243 patients, only 17.6% (95% CI: 12.3–24.5%) had a prevalence of any gastrointestinal symptom. However, SARS-CoV-2 RNA could be detected in either anal swabs or stool specimens in 29–80% of the tested patients (Cheung et al., 2020). It seems that infection in the gut occurs in a large percentage of patients, but the development of gastro-intestinal symptoms is less frequent.

## SARS-CoV-2 and the Human Gut Microbiome

The first pilot studies with small numbers of COVID-19 patients have found an altered microbiome compared to healthy controls (Gu et al., 2020; Zuo et al., 2020a,b; Chen et al., 2021; Yeoh et al., 2021; Zuo et al., 2021). These changes are also different from the changes seen in patients that were infected with the flu strain H1N1. There are limitations of these studies in terms of representation of age, sex, illness severities, comorbidities, ethnicities, diverse patient treatment (especially antibiotic treatment), severity of immune reaction during illness, diet, and heterogeneity of microbiome across the population. In our opinion, there is an urgent need to study the effect of SARS-CoV-2 on the human microbiome, especially the long-term effects, in larger trials and in more depth. However, besides these limitations, all studies reported a significant decrease in diversity and abundance and consequently an enrichment of opportunistic pathogens, also independently of antibiotic treatment—for example, one study showed the increased relative abundance of opportunistic pathogens like *Streptococcus* and *Rothia* (Gu et al., 2020). In a pilot study, Zuo et al. investigated the changes of the human gut microbiota during the time of hospitalization (Zuo et al., 2020b). Compared to healthy individuals, where *Eubacterium, Faecalibacterium prausnitzii, Roseburia*, and *Lachnospiraceae* taxa are prevalent, the gut microbiome of patients with COVID-19 showed an enrichment of opportunistic pathogens—for example, *Clostridium hathewayi, Bacteroides nordii*, and *Actinomyces viscosus*—and, at the same time, depletion of commensals. These changes occurred for both patients that did and did not receive antibiotics. Most importantly, gut dysbiosis persists during the COVID-19 disease course, even after clearance/recovery from SARS-CoV-2 infection. The baseline fecal abundance of the bacteria *Coprobacillus, Clostridium ramosum*, and *Clostridium hathewayi* showed a strong correlation with COVID-19 disease severity. In contrast, *Alistipes onderdonkii* and *Faecalibacterium prausnitzii*, the later known to have an anti-inflammatory activity, showed an inverse correlation.

In another small study, Zuo et al. demonstrated that SARS-CoV-2 replication occurs even without any gastrointestinal symptoms (Zuo et al., 2021). This study confirmed that COVID-19 patients showed an enrichment of opportunistic pathogens coupled with a loss of salutary bacteria. In this study, the authors reported that fecal samples with high SARS-CoV-2 infectivity had higher abundances of bacterial species *Collinsella aerofaciens, Collinsella tanakaei, Streptococcus infantis*, and *Morganella morganii*. Functional profiling revealed that these bacteria increase the capacity for biosynthesis of nucleotide and amino acid and carbohydrate metabolism (glycolysis). In contrast, fecal samples with signature of low to no SARS-CoV-2 infectivity had a higher abundance of *Parabacteroides merdae, Bacteroides stercoris, Alistipes onderdonkii*, and *Lachnospiraceae bacterium 1_1_57FAA*. Functional profiling revealed increases in the capability to produce short-chain fatty acids.

Gou et al. have discovered blood proteomic biomarkers that can predict the severity of COVID-19 (Gou et al., 2020). Gut microbial features like the relative abundance of *Bacteroides* genus, *Streptococcus* genus, *Lactobacillus* genus*, Ruminococcaceae* family, *Lachnospiraceae* family, and *Clostridiales* order will drive these biomarkers. The fecal metabolome was investigated and showed that 45 fecal metabolites, mainly within the categories of amino acids, fatty acids, and bile acids, can provide a link between the identified core gut microbiota, inflammation, and COVID-19 susceptibility.

The human gut mycobiome has also shown to be influenced by infection with SARS-CoV-2 (Zuo et al., 2020a). Hospitalized COVID-19 patients show a more heterogeneous mycobiome than the healthy control group. That indicates a transition into a more unstable microbial community. Patients had increased proportions of opportunistic fungal pathogens—*Candida albicans, Candida auris*, and *Aspergillus flavus*—compared with the controls.

A study with 70 hospitalized COVID-19 patients, where 28 got a multi-strain probiotic, showed a clear evidence of benefit for the patients (d'Ettorre et al., 2020). All patients who received the probiotic showed a higher reduction in diarrhea and other symptoms and also an eightfold lower risk in developing respiratory failure, a lower prevalence to be transferred to ICU, and a lower mortality rate. A similar study comprising 200 patients with severe COVID-19 pneumonia, where 88 got an additional multi-strain probiotic, also showed 19% reduced mortality in the probiotic-treated group (Ceccarelli et al., 2021).

## Is There a Connection Between Chronic COVID-19 Syndrome and Changes in Human Gut Microbiome?

There is evidence from several studies that SARS-CoV-2 infection leads to changes in the microbiome. These changes can be caused by an infection directly in the gut, as a response to increased inflammation and crosstalk between the oral, lung, and gut microbiome. In case a dysbiosis in the microbiome is established, it can lead to or fuel inflammation, increase intestinal permeability, and change the balance of signaling metabolites. In addition, there is a complex interplay of gene expression regulation *via* miRNA produced by the host, microbiomes, and SARS-CoV-2 (Hong and Kim, 2021; Omer and Kubra, 2021).

We observed that some of the symptoms described in CCS, like fatigue, sleep disturbance, joint pain, anxiety/depression, headache, and diarrhea, have also been correlated with a dysbiosis of the gut microbiome (Galland, 2014; Poroyko et al., 2016; Molina-Torres et al., 2019; Peirce and Alviña, 2019; Smith et al., 2019; Arzani et al., 2020; Matenchuk et al., 2020; Ogawa et al., 2020). In our opinion, the similarities of symptoms are very high, and given the fact that dysbiosis has been shown as a consequence of SARS-CoV-2 infection, a connection between CSS and dysbiosis of the microbiome should be considered for further research despite the fact that there is no direct evidence for this link yet. There are, however, initial positive effects by using probiotics on acute COVID-19.

Here we postulate that, in a subset of patients, long-term changes (dysbiosis) in the gut microbiota might drive or support some symptoms, especially fatigue, joint pain, diarrhea, headache, depression, and anxiety, as seen in chronic COVID-19 syndrome. Dysbiosis in the gut microbiome can influence the immune system, lung, and brain *via* the gut–lung axis and gut–brain axis as well as other organs *via* miRNA and metabolites produced by the microbiome. The gastrointestinal tract has not just a digestive function but also is responsible for achieving an immune system homeostasis. Yeoh et al. pointed out that dysbiosis seen in COVID-19 patients drives inflammation and fuels long-term symptoms (Yeoh et al., 2021). More research needs to be done to investigate this possible correlation between gut microbiome and CSS. In case such a correlation can be found in a subgroup of patients, treatment can be initiated by nutritional changes, pre- and probiotic supplements, or fecal transplants.

## Author Contributions

KH contributed to conceptualization, writing—original draft preparation, resources, and writing—review and editing. RM contributed to writing—review and editing, resources, and funding acquisition. Both authors have read and agreed to the published version of the manuscript.

## Conflict of Interest

The authors declare that the research was conducted in the absence of any commercial or financial relationships that could be construed as a potential conflict of interest.

## Publisher's Note

All claims expressed in this article are solely those of the authors and do not necessarily represent those of their affiliated organizations, or those of the publisher, the editors and the reviewers. Any product that may be evaluated in this article, or claim that may be made by its manufacturer, is not guaranteed or endorsed by the publisher.
